# Case Report: A case of synchronous multiple early gastric cancer with a microsatellite instability-high phenotype

**DOI:** 10.3389/fonc.2025.1527495

**Published:** 2025-04-03

**Authors:** Xinshuo Wang, Yifan Zhang, Guangyan Fan, Honglei Wu, Xing Qi, Xiujie Cui, Chengjun Zhou

**Affiliations:** ^1^ Cheeloo College of Medicine, Shandong University, Jinan, Shandong, China; ^2^ Department of Gastroenterology, The Second Hospital of Shandong University, Cheeloo College of Medicine, Shandong University, Jinan, Shandong, China; ^3^ Department of Pathology, The Second Hospital of Shandong University, Cheeloo College of Medicine, Shandong University, Jinan, Shandong, China

**Keywords:** SMEGC, MSI-H, BRAF mutation, immunotherapy, case report

## Abstract

Synchronous multiple early gastric cancer (SMEGC) is a relatively uncommon variant of early gastric cancer (EGC). In this report, we present a case of SMEGC accompanied by a microsatellite instability-high (MSI-H) phenotype. The patient was a 69-year-old man who presented to our hospital with abdominal pain. The endoscopic examination revealed two lesions. Both lesions were pathologically confirmed as EGC, then the patient subsequently underwent endoscopic submucosal dissection (ESD). Nine months post-procedure, the patient returned with recurrent abdominal pain, leading to the diagnosis of a new EGC. Immunohistochemical analysis demonstrated that all lesions exhibited an MSI-H phenotype and BRAF mutant expression, suggesting that these lesions are not associated with Lynch syndrome-related EGC. The case was ultimately diagnosed as SMEGC with an MSI-H phenotype. The current evidence and clinical experience suggest that patients with advanced MSI-H are likely to benefit from immunotherapy and should be considered for early systemic treatment with immunotherapy as a central component. At present, research studies on the molecular characteristics of SMEGC are limited, underscoring the importance of conducting comprehensive molecular diagnostics of each EGC patient, which could help clinicians thoroughly understand the lesion’s characteristics.

## Introduction

Gastric cancer (GC) ranks fifth in terms of both incidence and mortality among all cancers globally (1). Gastric adenocarcinoma is the predominant histological type of GC (2). According to the World Health Organization (WHO) classification, GCs are categorized into major histological types: tubular, papillary, mucinous, poorly cohesive, and mixed GC.

The Cancer Genome Atlas (TCGA) further classifies GCs into four molecular subtypes: Epstein-Barr virus-positive (EBV+), microsatellite instability (MSI), genomically stable (GS), and chromosomal instability (CIN). DNA mismatch repair (MMR) is a critical mechanism responsible for identifying and correcting base-pairing errors that arise during DNA replication and genetic recombination, thereby maintaining the stability of genetic information. This process involves a series of DNA MMR genes, including *MLH1*, *MLH3*, *MSH2*, *MSH3*, *MSH6*, *PMS1*, and *PMS2* (3, 4). Among these, *MLH1*, *PMS2*, *MSH2*, and *MSH6* are considered the most crucial MMR genes. The MMR system primarily functions through the formation of heterodimers, such as MutLα (MLH1-PMS2) and MutSα (MSH2-MSH6), with MLH1 and MSH2 playing pivotal roles. MSI refers to the hypermutation phenotype of genomic microsatellites that occurs when the MMR mechanism is defective (5–7). The degree of MSI can be categorized into microsatellite instability-high (MSI-H), microsatellite instability-low (MSI-L), and microsatellite stable (MSS) (8, 9). Immunohistochemical staining is the most commonly employed method to assess the expression of the four MMR proteins in tumor specimens. If all four proteins are positively expressed, the sample is considered mismatch repair-proficient (pMMR). Conversely, if any of the proteins are negatively expressed, the sample is deemed mismatch repair-deficient (dMMR). Typically, dMMR and pMMR correspond to the MSI-H phenotype and the MSI-L/MSS phenotype, respectively.

Early gastric cancer (EGC) is characterized by cancerous tissue confined to the mucosa or submucosa, with or without lymph node metastasis. Currently, endoscopic submucosal dissection (ESD) has supplanted traditional laparotomy as the preferred treatment for EGC and precancerous lesions. In cases of non-metastatic EGC, ESD not only facilitates the removal of the lesion but also preserves the normal anatomical structure and physiological function of the organ. This approach significantly enhances the quality of life for patients, offering advantages such as minimal trauma, rapid recovery, fewer complications, and promising efficacy (10).

Multiple early gastric cancer (MEGC) is defined according to Moertel’s criteria as follows (11): (1) each lesion is pathologically confirmed as malignant; (2) each lesion is distinctly separated from the others by normal gastric wall tissue, as verified microscopically; (3) the possibility that any lesion represents a metastatic tumor or local extension must be excluded. Furthermore, it is essential to distinguish between the main and minor lesions in cases of MEGC. In cases where the depth of invasion of lesions is equivalent, the lesion with the largest diameter is designated as the main lesion, also referred to as the major lesion. Conversely, if the lesions exhibit varying depths of invasion, the one with the greatest depth is identified as the main lesion. In instances where there are more than three EGC lesions, the secondary main lesion is regarded as the minor lesion, also known as the accessory lesion (12). Synchronous multiple early gastric cancer (SMEGC) is characterized by the presence of two or more early cancer lesions concurrently or by EGC lesions identified at different sites within 12 months post-surgery during follow-up (13). Research indicates that the rate of missed diagnosis for SMEGC ranges from 19% to 27% (14, 15).

In this report, we present a case of SMEGC exhibiting an MSI-H phenotype, where the MMR proteins MLH1 and PMS2 were negatively expressed, while MSH2 and MSH6 were positively expressed. Additionally, the lesion demonstrated a BRAF mutation. This case is presented to underscore the importance of clinicians remaining vigilant for the possibility of SMEGC in patients following ESD. Consequently, rigorous follow-up is essential to prevent missed diagnoses and misdiagnoses.

## Case report

On 18 October 2021, a 69-year-old male patient was admitted to the Gastroenterology Department of our hospital with abdominal pain. His medical history included elevated blood glucose levels and alcohol consumption, but no history of smoking. During white light endoscopy, two rough mucosal areas were identified in the lesser and greater curvatures of the gastric antrum, respectively. Upper endoscopy confirmed the presence of SMEGC.

**Figure 1:**
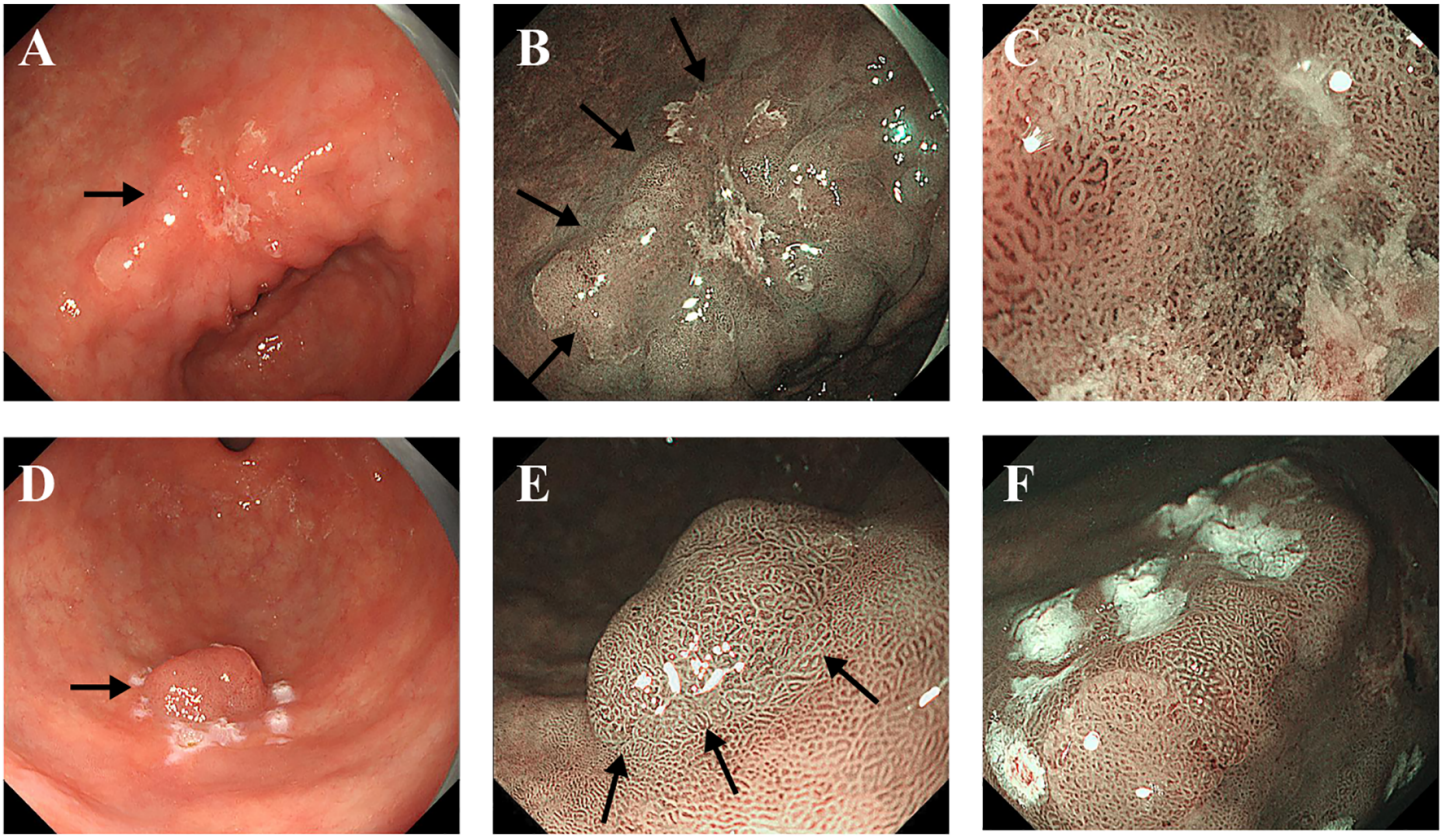
Endoscopic images of the major lesion **(A-C)** and the minor lesion **(D-F)**. **(A)** The white light endoscopic examination revealed a rough, red, slightly elevated, depressed lesion in the lesser curvature of the gastric antrum (Black arrow). **(B)** M-NBI showed an irregular microvascular pattern and a well-defined boundary (Black arrows). **(C)** The NBI showed the small and dense vessels with an epithelial circle (VEC) pattern. **(D)** The white light endoscopic examination revealed a rough, red, protruding lesion in the greater curvature of the gastric antrum (Black arrow). **(E)** M-NBI showed an irregular microvascular pattern and a well-defined boundary (Black arrows). **(F)** The size, morphology, and directionality of the glandular duct structure were inconsistent.

In the lesser curvature of the gastric antrum, a lesion characterized by rough redness, slight elevation and depression, measuring 4.0 cm ×3.0 cm, was observed (**Figure 1A**). Narrow-band imaging with magnification (M-NBI) revealed a well-defined boundary, atypical glandular ducts, and micro-vessels (**Figure 1B**). The lesion exhibited a pattern of small, dense vessels with an epithelial circle (VEC), and the micro-vessels appeared thickened and tortuous, with a clear demarcation from the surrounding tissues (**Figure 1C**). Additionally, a rough-redness protruding lesion measuring 1.5×1.5 cm was detected on the greater curvature of the gastric antrum (**Figure 1D**). The M-NBI also demonstrated a well-defined boundary for this lesion (**Figure 1E**). The size, morphology, and orientation of the glandular duct structures were found to be inconsistent (**Figure 1F**). The histopathological examinations of both lesions revealed a diagnosis of well-differentiated tubular adenocarcinoma (TAC) and papillary gastric adenocarcinoma (PGA). The patient subsequently underwent ESD at our institution.

**Figure 2:**
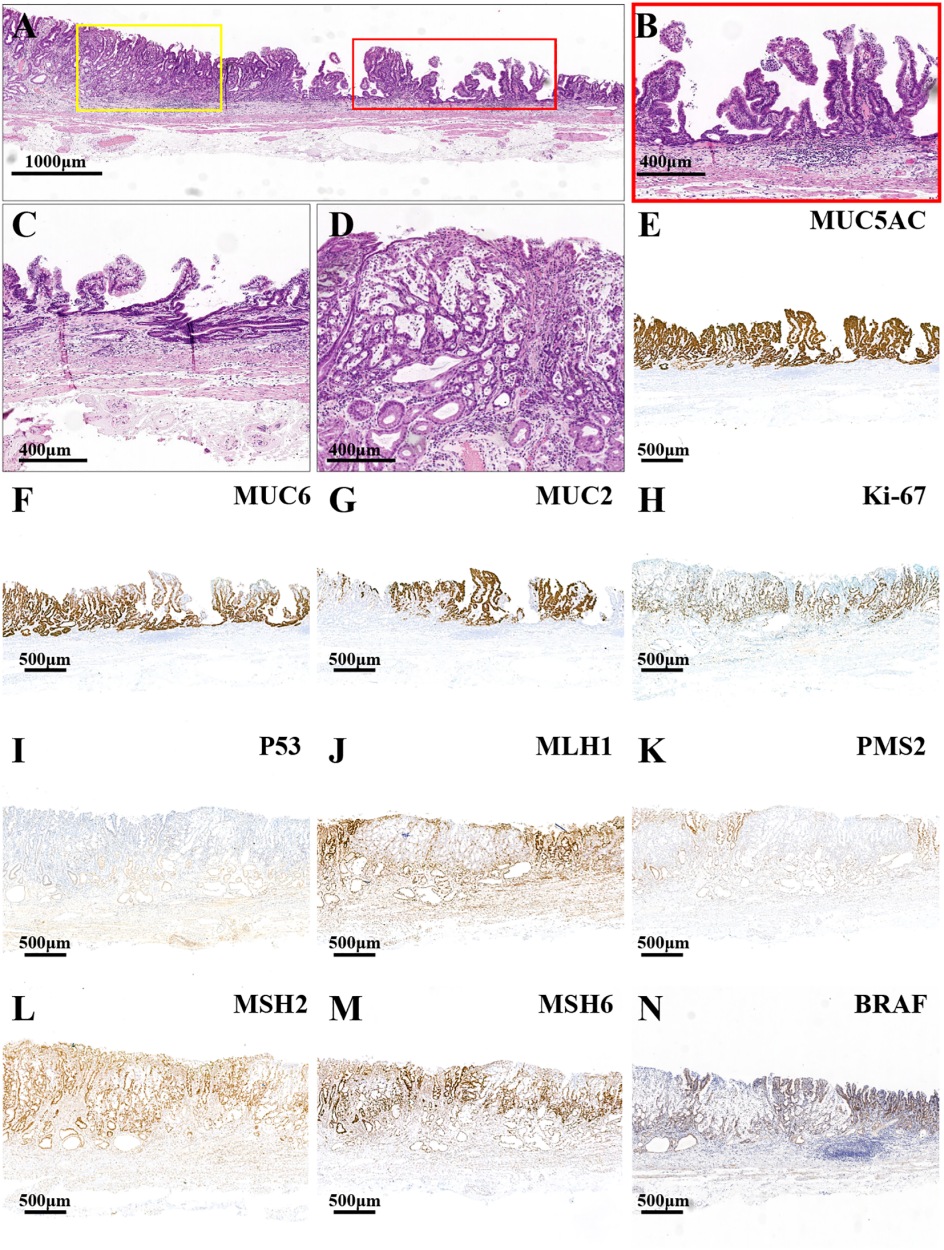
HE and immunohistochemistry staining images of the major lesion. **(A)** The red frame and the yellow frame indicate the papillary structure and tubular structure, respectively (the scale bar represents 1,000μm). **(B)** The high-power field images of the yellow frame in **(A)** the tumor cells displayed significant architectural atypia and low-grade cellular atypia in the surface papillary structure area (the scale bar represents 400μm). **(C)** The glandular duct fusion of the deep tubular tumors with a crawling growth pattern (the scale bar represents 400μm). **(D)** The focal poorly differentiated mucinous adenocarcinoma (the scale bars represent 400μm). **(E)** The lesion had a diffuse, strongly positive expression for MUC5AC. The lesion had a partially positive expression for MUC6 **(F)** and MUC2 **(G)**. **(H)** The index of Ki-67 was high (70%). **(I)** The lesion had a wild-type expression of P53. The mismatch repair (MMR) proteins MLH1 **(J)** and PMS2 **(K)** were negatively expressed, while MSH2 **(L)** and MSH6 **(M)** were positively expressed. **(N)** The lesion displayed BRAF mutant expression (the scale bars represent 500μm).

The main lesion, located in the lesser curvature of the gastric antrum, was histologically characterized by papillary and tubular structures confined to the mucosal layer (**Figure 2A**). In the area with a papillary structure on the surface, the tumor cells demonstrated significant architectural atypia and low-grade cellular atypia (**Figure 2B**). The deeper tubular tumors exhibited pronounced structural and cellular atypia, displaying glandular duct fusion with a crawling growth pattern (**Figure 2C**). Additionally, a small focal area of poorly differentiated mucinous adenocarcinoma was identified within the TAC (**Figure 2D**), where tumor cells were sporadically dispersed within mucin pools, displaying marked cellular atypia. The definitive pathological diagnosis of the main lesion was intramucosal PGA and moderately differentiated TAC with small foci of poorly differentiated mucinous adenocarcinoma. There was no evidence of vascular or lymphatic invasion. Ulceration was observed at the biopsy scar site, and both the horizontal and vertical margins were negative. The surrounding mucosa exhibited chronic gastritis with severe intestinal metaplasia and atrophy (type 0-IIa+IIc, 3.4cm×1.9cm, pT1a-M, Ly0, V0, pHM0, pVM0). Immunohistochemical staining revealed diffuse strong positivity for MUC5AC (**Figure 2E**) and partial positivity for MUC6 (**Figure 2F**) and MUC2 (**Figure 2G**). The Ki-67 proliferation index was elevated at 70% (**Figure 2H**), and the lesion exhibited a wild-type expression pattern for P53 (**Figure 2I**). Additionally, the MMR proteins MLH1 (**Figure 2J**) and PMS2 (**Figure 2K**) were not expressed, whereas MSH2 (**Figure 2L**) and MSH6 (**Figure 2M**) were expressed, indicating an MSI-H phenotype. Furthermore, the lesion demonstrated a BRAF mutation (**Figure 2N**).

**Figure 3:**
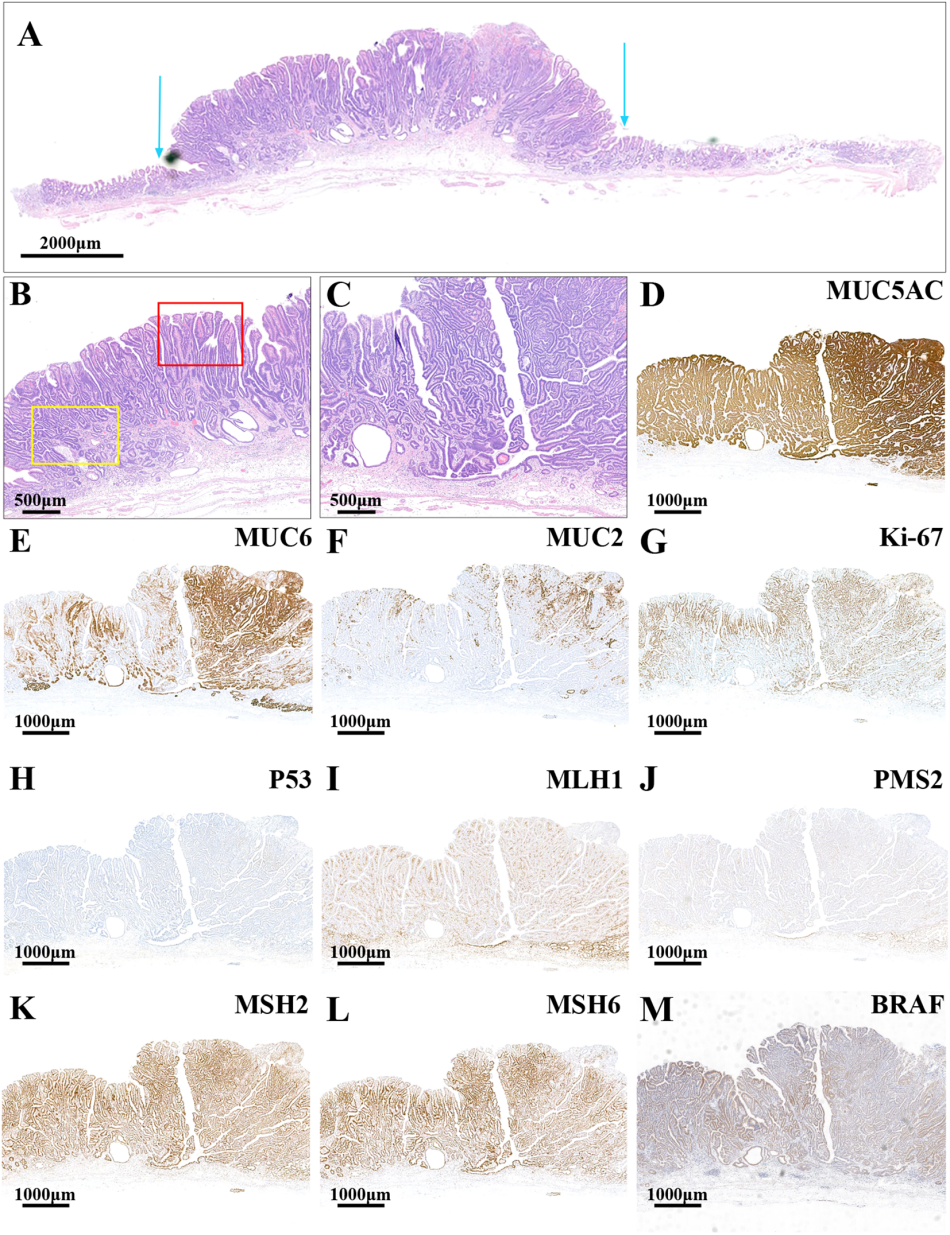
HE and immunohistochemistry staining images of the minor lesion. **(A)** The lesion had an obvious architectural atypia and low-grade cellular atypia. The blue arrows show the boundaries of the tumor (the scale bar represents 2,000μm). **(B)** The superficial structures of the lesion had a complex composition: branching, elongated villiform and papillary structures (the red frame) were found in the lesion, and slit-like serrations and ectopic glandular ducts were also revealed (the yellow frame; the scale bar represents 500μm). **(C)** The glandular ducts were fused, showing a crawling growth pattern (the scale bar represents 500μm). **(D)** The lesion had a diffuse strongly positive expression of MUC5AC but a partially positive expression of MUC6 **(E)** and MUC2 **(F)**. **(G)** The index of Ki-67 was high (50%). **(H)** The lesion had a wild-type expression of P53. The MMR proteins MLH1 **(I)** and PMS2 **(J)** were negatively expressed, while MSH2 **(K)** and MSH6 **(L)** were positively expressed. **(M)** The lesion had BRAF mutant expression (the scale bars represent 1,000μm).

The minor lesion located in the greater curvature of the gastric antrum exhibited morphological similarities to the main lesion. The lesion demonstrated pronounced architectural atypia and low-grade cellular atypia (**Figure 3A**). The lesion’s surface was characterized by complex, branching, elongated villiform and papillary structures with terminal enlargement. Additionally, slit-like serrations and ectopic glandular ducts were observed (**Figure 3B**). The cytoplasm of certain tumor cells was eosinophilic and devoid of mucus, whereas others were clear and rich in mucin. The structural and cellular atypia of the deeper tumor layers were significant, with glandular ducts exhibiting fusion and a crawling growth pattern (**Figure 3C**). Pathologically, the minor lesion was diagnosed as intramucosal PGA and moderately differentiated TAC. There was no evidence of vascular or lymphatic invasion, and both the horizontal and vertical surgical margins were negative. The surrounding mucosa displayed chronic gastritis with severe intestinal metaplasia and atrophy (type 0-I, 1.2 cm×1.0 cm, pT1a-M, Ly0, V0, pHM0, pVM0). Immunohistochemical analysis revealed diffuse strong positive expression for MUC5AC (**Figure 3D**) and partial positive expression for MUC6 (**Figure 3E**) and MUC2 (**Figure 3F**). The Ki-67 index (**Figure 3G**) was elevated at 50%, and the lesion exhibited wild-type P53 expression (**Figure 3H**). Furthermore, there was a loss of MLH1 (**Figure 3I**) and PMS2 (**Figure 3J**) protein expression, while the lesion remained positive for MMR proteins MSH2 (**Figure 3K**) and MSH6 (**Figure 3L**), indicating an MSI-H phenotype. Additionally, the lesion was found to harbor a BRAF mutation (**Figure 3M**).

**Figure 4:**
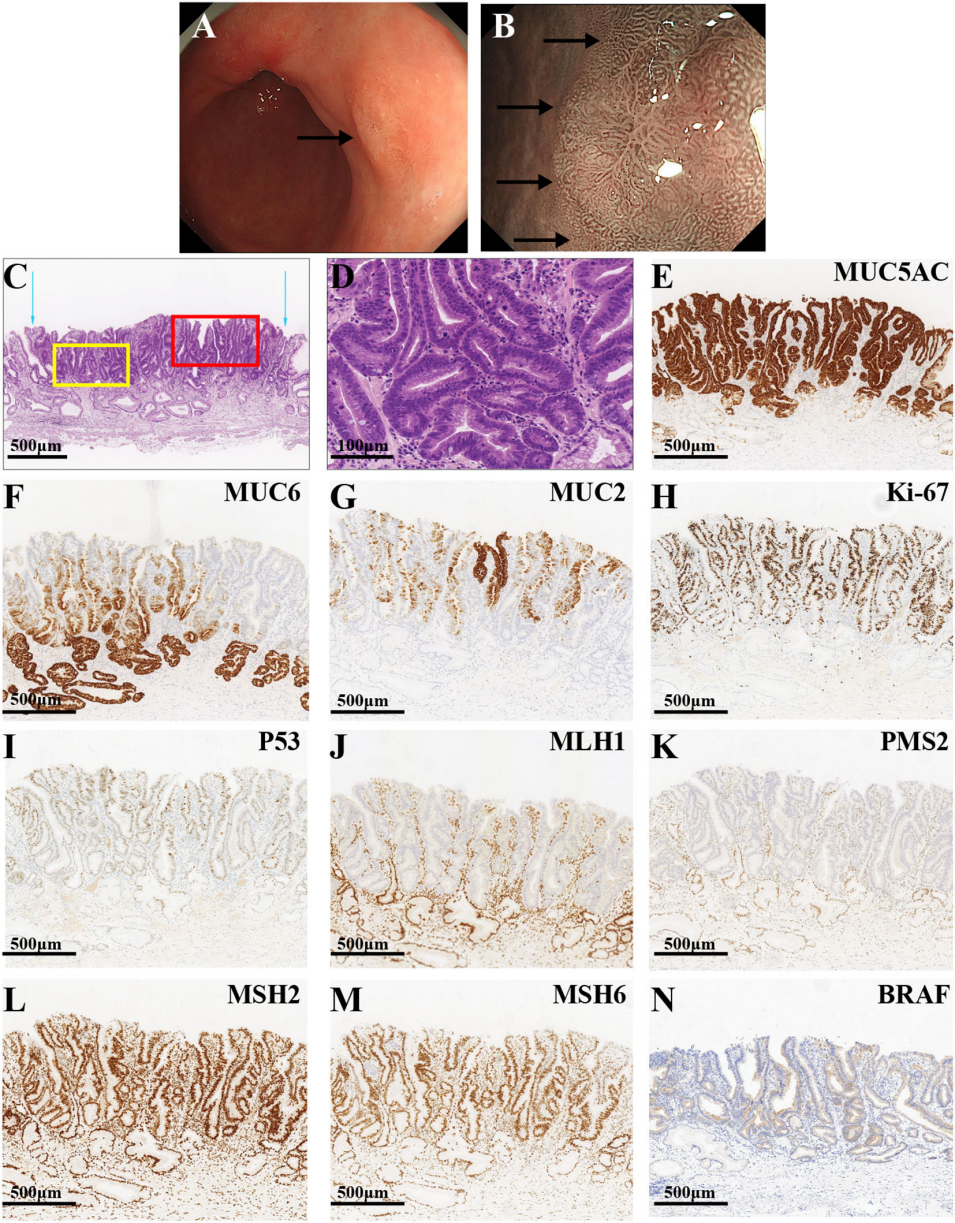
The endoscopic, HE, and immunohistochemistry staining images of the third lesion. **(A)** The white light endoscopic examination revealed a patchy, rough, well-defined, slightly elevated lesion on the posterior wall of the gastric antrum (black arrow). **(B)** The M-NBI showed a slightly elevated lesion with a well-defined boundary (black arrows). **(C)** The blue arrows show the boundaries of the tumor. The red frame and yellow frame indicate the superficial papillary structures and the deeper glandular tubular structures, respectively (the scale bar represents 500μm). **(D)** The tumor cells exhibited a slightly increased nucleo-cytoplasmic ratio, undisturbed nuclear polarity, eosinophilic cytoplasm, and the lack of mucus with remarkable architectural atypia and low cellular atypia (the scale bar represents 100μm). **(E)** The lesion had a diffuse, strongly positive expression of MUC5AC, with a partially positive expression of MUC6 **(F)** and MUC2 **(G)**. **(H)** The index of Ki-67 was high (70%). **(I)** The lesion had a wild-type expression of P53. The MMR proteins MLH1 **(J)** and PMS2 **(K)** were negatively expressed, while MSH2 **(L)** and MSH6 **(M)** were positively expressed. **(N)** The lesion had BRAF mutant expression (the scale bars represent 500μm).

The gastroenterologist advised the patient and his family members to return to the hospital for follow-up visits 3 and 6 months after the operation, but the patient did not return to the hospital on time as instructed by the doctor. Then, on 12 July 2022, 9 months after the ESD, the patient presented for re-treatment due to recurrent abdominal pain. White light endoscopy revealed a patchy, rough, well-defined, slightly elevated lesion measuring 1.0 cm ×1.0 cm on the posterior wall of the gastric antrum (**Figure 4A**). M-NBI demonstrated a well-defined boundary for the lesion. The size, morphology, and orientation of the glandular duct structures were inconsistent, which is consistent with a lesion located on the greater curvature of the gastric antrum (**Figure 4B**). Biopsy pathology confirmed a diagnosis of high-grade intraepithelial neoplasia (HGIN). Consequently, the patient underwent ESD treatment once more. Hematoxylin and eosin (HE) staining under microscopic examination revealed significant architectural atypia but low cellular atypia. The lesion’s surface demonstrated papillary structures, whereas the deeper layer exhibited partial differentiation towards tubular structures (**Figure 4C**). Tumor cells displayed a slightly elevated nucleo-cytoplasmic ratio, maintained nuclear polarity, eosinophilic cytoplasm, and an absence of mucus (**Figure 4D**). The definitive pathological diagnosis identified the lesion as intramucosal PGA and well-differentiated TAC. There was no evidence of vascular or lymphatic invasion, and both horizontal and vertical margins were negative. The surrounding mucosa exhibited chronic gastritis with severe intestinal metaplasia and atrophy (type 0-IIa, 0.6 cm×0.4 cm, pT1a-M, Ly0, V0, pHM0, pVM0). Immunohistochemical staining revealed diffuse strong positivity for MUC5AC (**Figure 4E**), along with partial positivity for MUC6 (**Figure 4F**) and MUC2 (**Figure 4G**). The Ki-67 index was notably high at 70% (**Figure 4H**), and the lesion exhibited a wild-type expression of P53 (**Figure 4I**). Additionally, MMR proteins MLH1 (**Figure 4J**) and PMS2 (**Figure 4K**) were negative, whereas MSH2 (**Figure 4L**) and MSH6 (**Figure 4M**) were positive, indicating an MSI-H phenotype. Furthermore, the lesion exhibited BRAF mutant expression (**Figure 4N**).

In conjunction with the preceding two diagnostic findings, this lesion was classified as a missed diagnosis type of SMEGC with an MSI-H phenotype. The findings of this study also suggest that SMEGC may share some common genetic alterations, potentially through shared oncogenic pathways.

## Discussion

We present a case of SMEGC with an MSI-H phenotype, noting that the occurrence of three cancerous lesions in a patient with SMEGC is relatively rare. Moreover, all the lesions in this case showed distinct histological features, MSI-H phenotype, and BRAF mutant expression. This report supports the hypothesis that SMEGC may share certain genetic alterations and possibly a common carcinogenic pathway.

Previous studies have identified risk factors for SMEGC, including advanced age, male gender, *Helicobacter pylori* infection, severe intestinal metaplasia and atrophy, and depth of invasion (16). Tumor diameter and submucosal infiltration have been identified as the independent risk factors for MEGC (14, 17). Research has shown that *H. pylori* infection is present in 78.9% of patients with SMEGC, and *H. pylori* infection is closely associated with the occurrence of SMEGC (18, 19). Studies have revealed that under the background of *H. pylori* infection, SMEGC often exhibits the same MSI/MSS characteristics, and there are commonalities in the copy number variations of some representative tumor-suppressor genes. This suggests that *H. pylori* infection may play a role in the occurrence and development of SMEGC by influencing genetic alterations (20). Therefore, even after the *H. pylori* infection is treated, there is still a possibility of developing SMEGC. However, other studies hold the opposite view, which is consistent with our research (21, 22). In this case, *H. pylori* infection was not detected, indicating that *H. pylori* may not be the cause of SMEGC in this patient. Kim et al. (23) proposed the hypothesis of “field carcinogenesis” in the context of SMEGC, suggesting that the entire gastric mucosa shares a uniform genetic and environmental background and a comparable level of exposure to carcinogens. ESD is recognized as an ultra-minimally invasive surgical approach for EGC. However, the incidence of postoperative synchronous cancer following ESD is reported to be higher than that associated with traditional surgical methods. This phenomenon may be attributed to the fact that ESD preserves a greater amount of gastric mucosa compared to conventional surgery (24). Despite the removal of lesions during prior procedures, the underlying conditions of severe intestinal metaplasia and atrophy remain present. Studies have indicated that the risk of SMEGC in patients with severe intestinal metaplasia is 1.75 to 3.32 times greater than in those without intestinal metaplasia (22, 25). The case under consideration exhibited severe intestinal metaplasia and atrophy, which may contribute to the development of multiple GCs. The most prevalent gross type of SMEGC is the superficial elevated type, characterized as differentiated adenocarcinoma (20, 21, 26). Furthermore, there may be a correlation between the distribution and degree of differentiation of the main and minor lesions in SMEGC (24, 26). In this study, all three EGC lesions were located in the distal stomach and were histologically classified as differentiated carcinoma with PGA components.

Compared to patients with solitary GC, those with SMEGC exhibit an increased frequency of the MSI phenotype (27, 28). However, there is a significant gap in the current research on the molecular differences between SMEGC and solitary early gastric cancer (SEGC). A retrospective study analyzed the immunohistochemical expression of p53 mutation, epidermal growth factor receptor (EGFR), human epidermal growth factor receptor 2 (CerbB), and MMR proteins in SMEGC and SEGC, and the results showed that there was no significant difference in the expression of the above molecules between the two groups (29). The literature indicates that defects in the MMR system are implicated in the early pathogenesis of GC (30). During normal DNA replication, the MutLα heterodimer (comprising MLH1 and PMS2) identifies and binds to minor mismatched bases, while the MutSα heterodimer (comprising MSH2 and MSH6) facilitates the excision and subsequent *de novo* synthesis of correct DNA bases at the mismatch site. Deficiencies in MLH1 or MSH2 result in corresponding deficiencies in PMS2 or MSH6, respectively. In tumor cells, dysfunctional MMR proteins fail to correct DNA replication errors, leading to their accumulation throughout the genome and contributing to tumorigenesis. The frequency of MLH1 gene methylation was significantly higher in EGCs exhibiting the MSI-H phenotype compared to those with the MSI-L or MSS phenotypes (31). In this study, all three EGC lesions exhibited negative expression for MLH1 and PMS2, indicating a dMMR/MSI-H phenotype, which may contribute to the development of SMEGC. More investigations of the molecular differences between SMEGC and SEGC will provide a theoretical basis for a deeper understanding of the occurrence and development mechanism of the two types of EGC.

Lynch syndrome (LS) is a hereditary cancer syndrome characterized by high penetrance, resulting from germline mutations in MMR genes. Patients with LS face a significantly elevated risk of developing tumors across multiple organ systems (32), including colorectal, gastric, and endometrial cancers. In addition to germline mutations in MMR genes, individuals with LS frequently present with dMMR/MSI-H phenotypes (33). Statistically, approximately 85% to 92% of LS patients exhibit an MSI-H phenotype (34). The V-raf murine sarcoma viral oncogene homolog B (BRAF) is part of a family of serine/threonine protein kinases, with mutations in the BRAF gene primarily resulting in the substitution of valine for glutamic acid at nucleotide 600, known as the V600E mutation (35). Numerous studies have established a strong association between BRAF mutations and the MSI-H/dMMR phenotype, as well as with MLH1 methylation and the CpG island methylator phenotype (CIMP) (36–38). Furthermore, BRAF mutations are linked to the silencing of MLH1 through the hypermethylation of the hMLH1 promoter, which manifests as dMMR/MSI (39, 40). The constitutive activation of the MAPK signaling pathway, induced by BRAF mutations, can lead to abnormal cell proliferation and differentiation, ultimately resulting in tumorigenesis (35). However, unlike cases involving BRAF mutations, the majority of LS patients are BRAF wild-type, indicating a distinct mechanism of carcinogenesis (41). Consequently, BRAF mutations do not coexist with LS (42, 43). In this case, the lesions exhibited an MSI-H phenotype and BRAFV600E mutation expression, effectively excluding an LS diagnosis.

Research has demonstrated that MSI in GC is associated with advanced age, occurrence in the lower part of the stomach, mixed tissue types in cancer, and the presence of multiple cancers. From a histogenetic perspective, GC with MSI predominantly manifests as differentiated carcinoma, with some cases progressing to solid poorly differentiated adenocarcinoma (PDA) (44). Studies have indicated that MSI is more frequently observed in both PGA and PDA (45–47). Crawling-type gastric adenocarcinoma (CRA), essentially a moderately differentiated TAC, exhibits unique morphological characteristics. The biological properties of CRA are characterized by high invasiveness and a poor prognosis. In our case, all three lesions were accompanied by PGA and CRA, leading us to hypothesize that the mixed adenocarcinoma components may have resulted from MSI-H (31). MSI-H GC is associated with more aggressive tumor characteristics, including deeper local invasion and lymphovascular invasion (LVI), and shows a tendency towards increased lymph node (LN) metastasis. The prevalence of the MSI subtype of GC decreases with advancing pathological disease stage and is linked to a more favorable prognosis compared to MSS GC (48–50). However, current studies indicate that MSI status is not a prognostic marker in patients with EGC.

As research into precision therapy for tumors advances, immunotherapy and targeted therapy, in combination with traditional radiotherapy and chemotherapy, are demonstrating synergistic effects. MSI tumors are potentially more sensitive to immunotherapy due to their inherent mutational burden, heightened inflammatory response, and increased expression of immune checkpoints (51, 52). Deficiencies in MMR proteins result in defects in DNA replication, leading to the accumulation of mutations and the expression of neoantigens, which may serve as potential targets for immune cells (53). Moreover, tumors characterized by MSI-H/dMMR typically exhibit significant lymphocyte infiltration, which can potentiate the immune response of tumor infiltrating lymphocytes (TILs) and thereby enhance the efficacy of immune checkpoint inhibitors (ICIs). Additionally, MSI-H tumors frequently present with a high tumor mutation burden (TMB-H), a recognized marker of sensitivity to immunotherapy (54, 55). Classifying tumors based on MSI status offers a valuable approach to identifying patients who may benefit from immunotherapy or targeted therapy. Consequently, it is imperative that each patient with EGC undergo a comprehensive molecular diagnosis to thoroughly characterize the lesion.

Due to the rarity of SMEGC cases, there is a paucity of detailed clinical and pathological analyses. In this report, we presented a case of SMEGC with an MSI-H phenotype to contribute to the growing body of evidence, thereby alerting clinicians to the heightened risk of synchronous multiple cancers in high-risk patients following ESD. For postoperative patients with early-stage cancer, it is crucial to conduct systematic and meticulous endoscopic surveillance. It is imperative to not only focus on patients exhibiting moderate to severe atrophy and intestinal metaplasia within the background mucosa but also to give considerable attention to those with alterations in MMR protein status. This approach facilitates the timely detection of SMEGC, thereby reducing the risk of missed diagnoses and misdiagnoses, ultimately enhancing patient survival and prognosis. Furthermore, additional research is essential for healthcare professionals to refine treatment strategies and utilize MSI-H as a biomarker for patients with EGC to better identify SMEGC.

## Conclusion

SMEGC is a relatively rare form of EGC. We presented a case of SMEGC with an MSI-H phenotype, characterized by distinct histological and immunophenotypic features, to underscore the importance of vigilance among clinicians regarding the potential for SMEGC in patients post-ESD. Rigorous follow-up is crucial to prevent diagnostic oversights.

## Data Availability

The original contributions presented in the study are included in the article/**Supplementary Material**. Further inquiries can be directed to the corresponding authors.
